# Hepatic Abscess Secondary to a Retained Appendicolith Following Perforated Appendicitis: A Case Report

**DOI:** 10.7759/cureus.96618

**Published:** 2025-11-11

**Authors:** Jad Kabbara, Basel Diab, Mohamad Mouchli

**Affiliations:** 1 Anesthesiology, Lake Erie College of Osteopathic Medicine, Greensburg, USA; 2 Internal Medicine, Lake Erie College of Osteopathic Medicine, Erie, USA; 3 Gastroenterology, Fisher-Titus Medical Center, Norwalk, USA

**Keywords:** appendicitis, dropped appendicolith, giant appendicolith, hepatic abscesses, retained appendicolith

## Abstract

In rare cases, intra-abdominal abscesses can be caused by retained appendicoliths. Additionally, hepatic abscesses can also occur after dropped appendicoliths and are exceptionally rare. This report showcases a case of a 75-year-old man who developed a right subhepatic abscess five months after undergoing an appendectomy for perforated appendicitis. It was revealed through imaging that there was a peripherally enhancing fluid collection adjacent to the right lobe of the liver that contained a calcified appendicolith. Additionally, retrospective review of prior scans confirmed the presence of an appendicolith that was left intraperitoneally, which likely occurred after the appendix perforation or intraoperative spillage. After confirming the presence of the abscess, the patient was successfully treated with broad-spectrum antibiotics along with percutaneous drainage of the abscess. This case demonstrates the importance of understanding that dropped appendicoliths are a rare but potential etiology for abscesses in patients undergoing appendectomy. It also shows the importance of proper radiological and intraoperative evaluation to identify and remove these fragments. Early identification and removal of a retained appendicolith can prevent persistent infection, recurrent abscess formation, and severe complications such as sepsis.

## Introduction

Acute appendicitis is a common surgical emergency with a lifetime risk of approximately 6-7% [1,2]. Typically, appendicitis can be definitively cured with appendectomy; however, complications can always arise, namely, with perforated appendicitis. An appendicolith, also known as a fecalith, is a calcified composition that is deposited in the appendix lumen. It is typically found on imaging and around 30% of patients with suspected appendicitis [2,3].

The presence of appendicoliths is commonly associated with complicated appendicitis and can be seen as a risk factor for failure of non-operative management. During surgical removal, appendicoliths can migrate into the peritoneal cavity and go unnoticed; this is what is known as a dropped appendicolith [3]. When an appendicolith is retained, it can cause infection, which leads to abscess formation in atypical locations. The most common location that they can cause abscesses is in the right lower quadrant or the pelvis. In some cases, they may migrate to the subhepatic area or within the liver, which can cause hepatic or perihepatic abscess.

Pyogenic liver abscess can be caused by appendicitis; however, only around 1% of pyogenic liver abscesses are caused by appendicitis [4,5]. In most cases, hepatic abscesses are caused due to portal venous seeding from a secondary, untreated infection. On the contrary, direct absence formation from a retained appendicolith is an atypical mechanism that has rarely been documented in case reports [4]. This is a presentation of a rare case where a dropped appendicolith caused a subhepatic abscess. We also review the relevant literature on this rare occurrence.

## Case presentation

A 75-year-old man with a past medical history significant for hypertension and a recent appendectomy for perforated acute appendicitis (six months prior) presented to his primary care physician with a five-month history of persistent right upper quadrant (RUQ) abdominal pain. The pain was dull and intermittent, without a clear precipitant. He denied fever, chills, or jaundice, and his bowel habits were normal.

**Figure 1:**
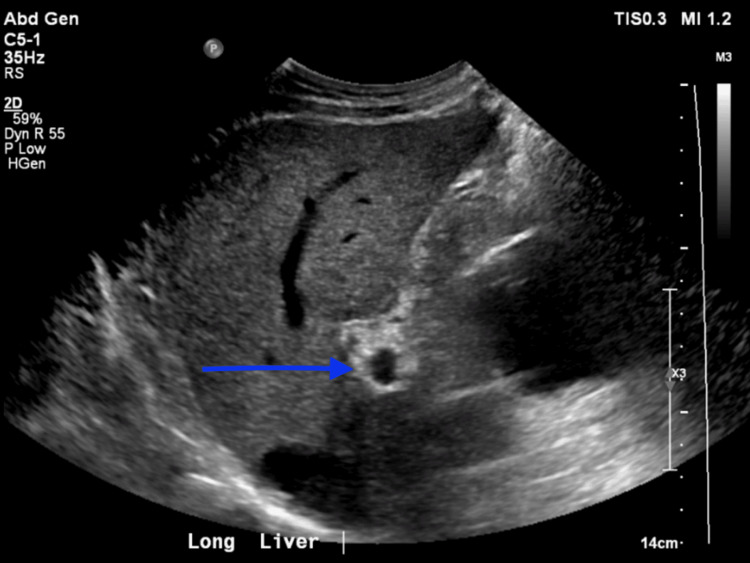
Right upper quadrant ultrasound demonstrating a cystic lesion at the inferior aspect of the right hepatic lobe (subcapsular region). The lesion appears anechoic with well-defined borders and no solid components, initially interpreted as a simple hepatic cyst.

**Figure 2:**
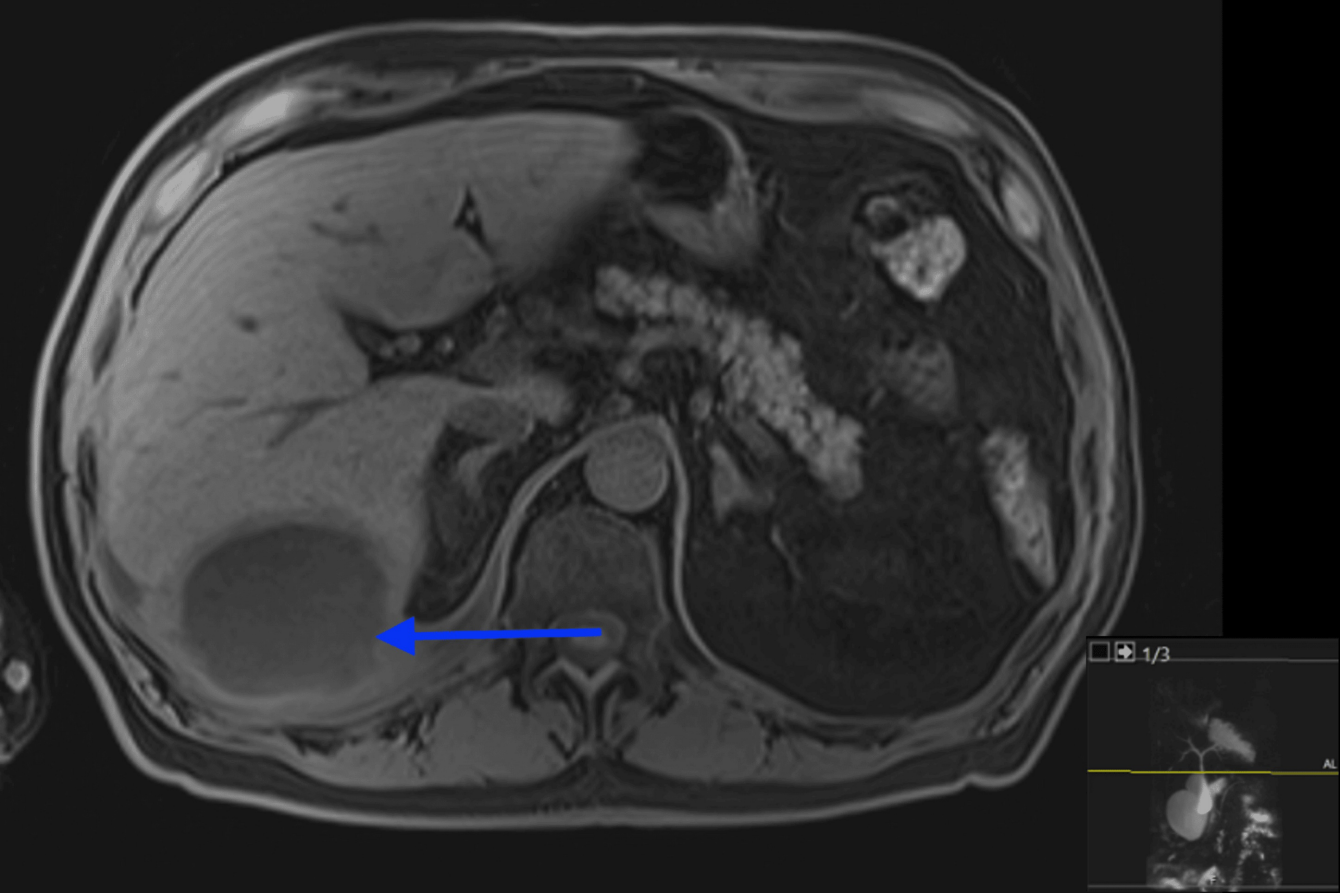
Contrast-enhanced MRI of the abdomen showing a rim-enhancing fluid collection posterior to the right hepatic lobe. The peripheral enhancement and adjacent hepatic capsule involvement are consistent with a localized subhepatic abscess.

Physical examination revealed mild RUQ tenderness without peritoneal signs. Laboratory studies were notable for a moderately elevated C-reactive protein and erythrocyte sedimentation rate, but his white blood cell count was only mildly elevated at 11×10^9^/L. Liver enzymes were within normal limits. Given the chronicity of the RUQ pain, an abdominal ultrasound was obtained, which showed a 2.8 cm cystic-appearing lesion at the inferior aspect of the right hepatic lobe (subcapsular region) with no solid components (Figure 1). This was initially interpreted as a possible simple liver cyst. However, due to the patient’s persistent symptoms, a follow-up contrast-enhanced magnetic resonance imaging (MRI) study was performed one month later. MRI of the abdomen revealed a rim-enhancing fluid collection posterior to the right hepatic lobe (segment VI/VII) measuring approximately 3 cm in diameter (Figure 2). This finding was consistent with a localized abscess adjacent to the liver capsule. The MRI did not clearly delineate any foreign body or calcification within the abscess, but the presence of peripheral enhancement and location raised suspicion for an unusual source of infection. 

**Figure 3:**
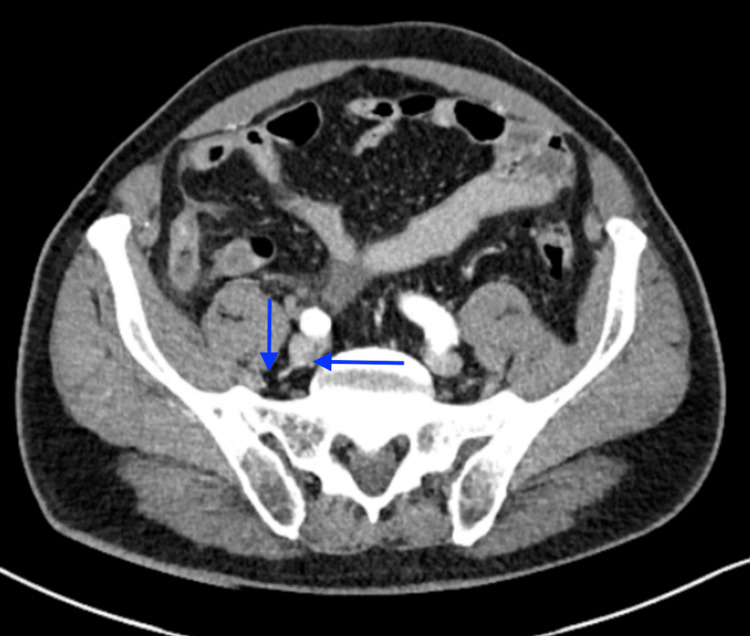
CT scan of the abdomen and pelvis performed six months earlier during the patient’s presentation with acute perforated appendicitis, demonstrating two calcified appendicoliths in the right lower quadrant, near the inflamed and perforated appendix.

**Figure 4:**
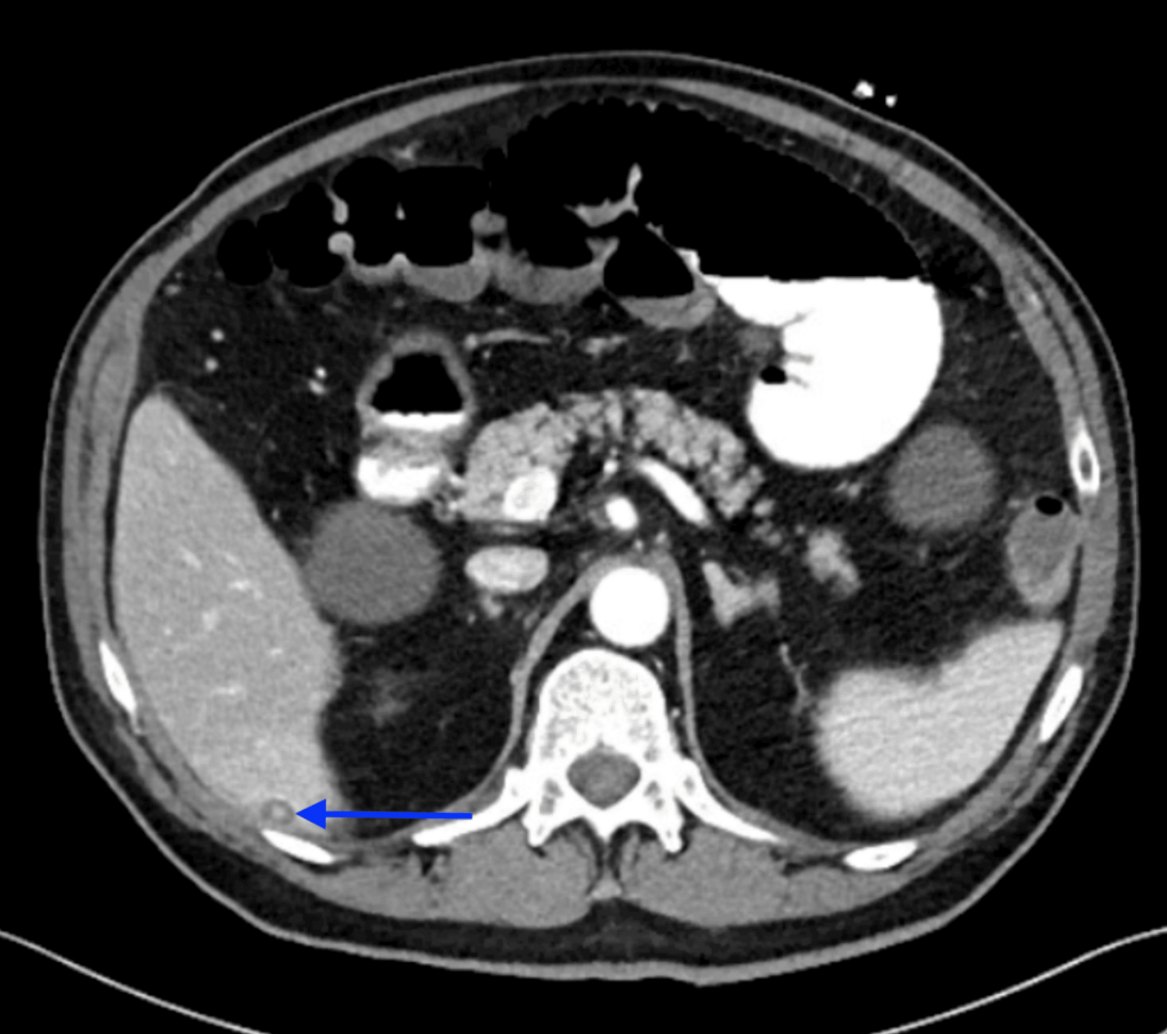
Post-operative CT scan obtained several weeks after appendectomy revealing a small round radiodensity along the posterior surface of the liver within the subhepatic space, retrospectively consistent with a retained appendicolith that later served as the nidus for abscess formation.

The patient’s history of a recent perforated appendicitis prompted a thorough review of his prior imaging. A computed tomography (CT) scan of the abdomen and pelvis obtained six months earlier, at the time of his appendicitis diagnosis, had demonstrated acute appendicitis with perforation and two calcified appendicoliths in the right lower quadrant (Figure 3). He had subsequently undergone an open appendectomy at that time. A post-operative CT scan obtained a few weeks after the appendectomy, conducted during the initial hospitalization due to persistent low-grade fevers, was re-evaluated. On this scan, we identified a small round radiodensity (~1 cm in size) along the posterior surface of the liver, in the subhepatic space, which had not been noted in the original report (Figure 4). This calcific density was new compared to the pre-operative imaging and was separate from the appendiceal region, indicating that one of the appendicoliths had migrated to the subhepatic region. In retrospect, the appendicolith likely escaped the appendiceal lumen during the perforation or was dropped during surgery and lodged near the liver.

Correlating the imaging findings and history, the diagnosis of a hepatic abscess secondary to a retained appendicolith was made. The patient was admitted for management. Broad-spectrum intravenous antibiotics were initiated, which consisted of coverage for gut flora such as Enterobacteriaceae and anaerobes. Interventional radiology was consulted, and an ultrasound-guided percutaneous drainage of the abscess was performed. Approximately 30 mL of purulent fluid was aspirated, and a pigtail catheter was left in place. Fluid culture grew *Escherichia coli*, consistent with an appendiceal source infection, and antibiotics were tailored to culture sensitivities. The calcified appendicolith itself was not retrieved during the drain placement; it was adherent in the subhepatic space and just abutting the liver capsule on imaging.

Given the patient’s clinical improvement and the risks of invasive surgery in an elderly patient, a decision was made to manage the appendicolith conservatively. The patient’s RUQ pain and inflammatory markers improved markedly over the next week. He had no complications from the drainage procedure. The drain was removed after one week when output had ceased, and a follow-up ultrasound showed collapse of the abscess cavity. A repeat contrast-enhanced CT scan one month after drainage confirmed complete resolution of the abscess with no fluid collection remaining. The previously seen calcific appendicolith was still visible on CT in the subhepatic space, but it appeared isolated and without surrounding fluid or inflammatory changes.

The patient remained asymptomatic at three-month follow-up on oral antibiotics, with no signs of recurrent infection. He was advised about the possibility of recurrence in the future, given the retained appendicolith, but he declined any further surgical intervention at that time. Unfortunately, while this case occurred over five years ago, additional long-term follow-up details are not available.

## Discussion

This case illustrates an unusual complication of a perforated appendicitis leading to the formation of a hepatic abscess due to a retained appendicolith. An appendicolith is a collection of fecal material and mineral deposits that form within the appendix and harden over time, leading to a “stone-like formation” [5,6]. Many times appendicolas are found incidentally on imaging and tend to be asymptomatic in many patients, with many patients never developing appendicitis. However, in the setting of acute appendicitis perforation, an asymptomatic appendicolith can be released from the appendix and make its way into the peritoneal cavity, becoming symptomatic [6]. Any spilled appendicolith that is not removed can act as a foreign body within the peritoneal cavity, leading to a breeding ground for infection, similar to how a spilled gallstone can form delayed abscess formation even after laparoscopic cholecystectomy. In a previous case report, serious retained appendicoliths were seen migrating to various locations within the abdomen or even crossing the diaphragm, leading to a plethora of intra- and extra-abdominal complications, which included intra-abdominal abscess, fistula formation, peritonitis, and even empyema due to the transdiaphragmatic migration [7]. The most common sites for abscess formation due to a dropped appendicolith include the pelvis, the subhepatic space between the liver and the kidney, and the right paracolic gutter.

Hepatic abscess formation due to appendicitis is a rare complication that was prevalent in the modern era of early surgery and antibiotics, due to uncontrolled appendicitis leading to portal vein thrombophlebitis and multiple liver abscesses. However, as antibiotic regimens have strengthened, appendicitis accounts now for a very small percentage of pyogenic liver abscess cases (less than 1%) [6,7]. What makes this case unique is the direct implantation of infection due to the retained appendiceal stone, of which, to our knowledge, there have been fewer than a dozen case reports of intraplastic abscess due to a dropped appendicolith in the literature. Previous case reports reporting appendicolith infection have been due to complications such as perforated appendicitis or complicated appendectomy, leading to the confirmed spillage of the appendicolith. Furthermore, the majority of patients in prior reports are relatively younger, ranging between 35 and 55 years in one literature review [8]; however, our patient, who was 75 years old, appears to be an outlier in terms of age, which highlights the uniqueness of this complication while although uncommon, can occur even in older adults [9,10]. It is also possible that the older population with this complication is less frequently reported due to other etiologies of the abscess formation that may overshadow a dropped stone.

Diagnosis of an appendicolith heavily relies on imaging, with ultrasound detecting an abscess and many times showing an echogenic focus with shadowing, suggesting a calcification [11]. Although ultrasound may help aid in diagnosis, CT tends to be the most effective modality for identifying appendicolith due to its high sensitivity for calcification. Appendicolith can usually appear as a high attenuation calcific density adjacent to an abscess cavity. In our specific case, the calcified appendicolith on CT aided in the confirmation of the source of infection. Although MRI can help show abscess anatomy and can be used to aid in characterizing liver lesions, many times, small calcifications can be missed. Our report shows how a chronic abscess from a dropped appendicolith, which was originally misdiagnosed until imaging eventually revealed the calcified mass, demonstrates how a missed finding on imaging led to a delayed diagnosis until the patient's symptoms persisted and previous scans were reviewed, allowing the etiology to become clear.

Management of appendicolith typically involves both removal of the abscess and the offending stone, with drainage of the abscess usually being achieved percutaneously or surgically. In many previously reported cases, percutaneous abscess drainage was performed to first stabilize the patients and clear the infection. In any case, simply draining the abscess while leaving the appendicolith in place is associated with a high risk of persistent infection and recurrent abscess formation. Thus, for definitive treatment, stone removal should be performed if feasible. That being said, the timing and method of appendicolith can vary, with some patients undergoing percutaneous retrieval with an interventional radiologist using tools such as snares or baskets to capture and remove the stone through the drainage track. However, other patients may require surgical intervention, either laparoscopically or open, to physically locate and remove the offending appendicolith, especially if the stone is larger or if previous percutaneous methods have failed [12]. In our patient, we elected to manage conservatively after percutaneous drainage, given his clinical Improvement and age. However, if the abscess had recurred or not fully resolved, surgical removal of the stone would have been indicated. Patients should always be counseled about the possibility of recurrence at the potential need for further interventions if an abscess were to reform.

Although rare, appendicolith complications can be severe, with chronic or recurrent intra-abdominal sepsis leading to adhesions, fistulous tracks, or even further extension of the infection to adjacent organs. Chronic abscess from a retained appendicolith can lead to sinus track formation in the abdominal wall through constant erosion [13]. Other complications that can occur are fistulization to the bladder or bowel and even extension through the diaphragm, leading to pleural empyema and pneumonitis. These complications further emphasize why a retained appendicolith should not be left to provoke further infection and should be properly managed.

## Conclusions

Hepatic abscess due to a retained appendicolith is an uncommon and clinically important complication that can develop due to appendicitis. This case report demonstrates that even many months after a perforated appendicitis and appendectomy, a dropped appendicolith can be a breeding ground for any source of infection in the abdominal cavity. Clinicians should maintain a high level of suspicion for retained appendicolith in patients who develop unexplained abscess formation following an appendectomy or patients who have chronic, persistent abdominal symptoms following a complicated appendicitis course.

Diagnosis and management include prompt imaging with a CT scan to carefully view calcifications in the abdominal cavity, and once identified, abscess drainage and, in most cases, removal of the appendicolith have been shown to prevent recurrence and properly treat the patient's symptoms. Minimal management with abscess drainage and antibiotics while leaving the stone can often lead to recurrent infection and further complications. Therefore, understanding this rare diagnosis is crucial for appropriate intervention to be undertaken in order to achieve full resolution. Our case report adds to the limited body of evidence on this topic and serves as a reminder that post-operative vigilance and adequate surgical technique are key to preventing and managing this rare complication of appendicitis.
